# Impact of different scanners and acquisition parameters on robustness of MR radiomics features based on women’s cervix

**DOI:** 10.1038/s41598-020-76989-0

**Published:** 2020-11-23

**Authors:** Honglan Mi, Mingyuan Yuan, Shiteng Suo, Jiejun Cheng, Suqin Li, Shaofeng Duan, Qing Lu

**Affiliations:** 1 Department of Radiology, Renji Hospital, School of Medicine, Shanghai Jiao Tong University, 160 Pujian Rd, Shanghai, 200127 China; 2 Department of Radiology, Affiliated Zhoupu Hospital, Shanghai University of Medicine & Health Sciences College, 1500 Zhouyuan Road, PongDong New District, Shanghai, 201318 China; 3 GE Healthcare China, Pudong new town, No1, Huatuo road, Shanghai, 210000 China

**Keywords:** Physics, Medical research

## Abstract

MR Radiomics based on cervical lesions from one single scanner has achieved promising results. However, it is a challenge to achieve clinical translation. Considering multi-scanners and non-uniform scanning parameters from different centers in a real-world medical scenario, we should first identify the influence of such conditions on the robustness of MR radiomics features (RFs) based on the female cervix. In this study, 9 healthy female volunteers were enrolled and 3 kiwis were selected as references. Each of them underwent T2 weighted imaging in three different 3.0-T MR scanners with uniform acquisition parameters, and in one MR scanner with various scanning parameters. A total of 396 RFs were extracted from their images with and without decile intensity normalization. The RFs’ reproducibility was evaluated by coefficient of variation (CV) and quartile coefficient of dispersion (QCD). Representative features were selected using the hierarchical cluster analysis and their discrimination abilities were estimated by ROC analysis through retrospective comparison with the junctional zone and the outer muscular layer of healthy cervix in patients (n = 58) with leiomyoma. This study showed that only a few RFs were robust across different MR scanners and acquisition parameters based on females’ cervix, which might be improved by decile intensity normalization method.

## Introduction

Radiomics offers a new way for tumor characterization in medical image analysis. Different from histological analysis, which is based on tissue samples obtained through biopsies and has difficulties to provide the full picture of the entire tumor, radiomics analysis is non-invasive and able to give insights into tumor heterogeneity^1^. During recent decades, the advances of medical imaging in hardware, standardized protocols, and improved methods facilitated the rapid development of radiomics and its combination with deep learning^2,3^. Increasing quantitative features are extracted from computed tomography (CT) and magnetic resonance imaging (MRI) to investigate tumor differential diagnosis, treatment response monitoring, prognosis, and prediction^4^. Until now, although radiomics studies of human have involved cervix, prostate, breast, brain and so on, most of them focused on data from one single scanner^5–10^. To achieve clinical translation, however, issues related to multi-scanners and non-uniform scanning parameters from different centers in a real-world medical scenario have to be first addressed.

Actually, not all the extracted features are reliable and reproducible even from one single scanner. Most of the radiomics features are affected not only by scanners, but also by acquisition parameters, such as field of view, spatial resolution, reconstruction algorithm, tube voltage (CT), and milliamperage (CT), repetition time (MR), echo time (MR)^11–13^. Compared with CT, MR is more complicated in nature and can be influenced by more acquisition parameters. Although a few studies have performed radiomics analysis using MR data based on non-uniform scanning parameters and different centers in human organs, such as brain and prostate, the reproducibility and reliability of radiomics features have not yet been systemically investigated^14–16^. Beyond that, different from brain and prostate, cervix is a relatively less stable organ considering that its shape and anatomical position could be affected by the filling status of bladder and rectum, and its MR signal intensity could also be influenced by the menstrual cycle. Therefore, we should first recognize the reproducibility of radiomics features influenced by different scanners and acquisition parameters before pooling multi-center data associated with cervical tissue to prospectively validate the value of radiomics from one single scanner.

T2 weighted imaging (T2WI) is a stable and essential sequence of cervical scanning according to the protocol for staging and evaluation of cervical cancer proposed by the European Society of Urogenital Radiology 2010^17^. Although radiomics analysis of cervical tissue has been widely performed on T2W images^6,18–20^, its non-quantitative nature underlines the need for investigating the reproducibility of this sequence in a multi-center scenario. Thus, the purpose of the current study was to quantitatively identify the influence of different scanners and acquisition parameters on the robustness of T2WI radiomics features (RFs) based on females’ cervix, which might have some implications for further radiomics studies on cervical lesions.

## Results

### Inter-MR analysis

The percentages of reproducible RFs obtained from three MR scanners are summarized in Table 1. Regarding the influence of the MR scanners on the robustness of RFs, reproducible RFs ranged from 51.5% (204 of 396) in G.0 (kiwis) to only 24.2% (96 of 396) in G.3 (volunteers) when using QCD and CV indexes with 15 and 0.1 as the cutoff values, respectively. After filtering based on CV < 0.1 for all kiwis and volunteers in G.0–G.3, only 23.5% (93 of 396) reproducible RFs were shared across all groups.

**Table 1:** The number of reproducible features for inter-MR analysis across volunteers and kiwis out of a total of 396.

Group	n	Cutoff Value	Number of reproducible features	Mean ± standard deviation
Kiwis (G.0)	3	CV < 0.1	124(31.3%)	0.28 ± 0.33
		QCD < 10	127(32.1%)	25.77 ± 26.02
		CV < 0.15	200(50.5%)	0.28 ± 0.33
		QCD < 15	204(51.5%)	25.77 ± 26.02
Volunteers (G.1)	3	CV < 0.1	123(31.1%)	0.50 ± 1.03
		QCD < 10	125(31.6%)	41.48 ± 65.75
		CV < 0.15	137(34.6%)	0.50 ± 1.03
		QCD < 15	139(35.1%)	41.48 ± 65.75
Volunteers (G.2)	3	CV < 0.1	98(24.8%)	0.36 ± 0.47
		QCD < 10	113(28.5%)	32.61 ± 41.93
		CV < 0.15	143(36.1%)	0.36 ± 0.47
		QCD < 15	144(36.4%)	32.61 ± 41.93
Volunteers (G.3)	3	CV < 0.1	96(24.2%)	0.72 ± 4.79
		QCD < 10	99(25.0%)	64.53 ± 313.22
		CV < 0.15	156(39.4%)	0.72 ± 4.79
		QCD < 15	157(39.7%)	64.53 ± 313.22

The values displayed on the table were means within each group. “Mean ± Standard Deviation” was calculated from mean CV or mean QCD values within each group. G.0 represents the three kiwis, while G.1–G.3 represent the three group volunteers in 6th–10th, 11th–15th, and16th–20th day of physiological cycle respectively.

### Intra-MR analysis

The percentages of reproducible RFs based on different scanning parameters are summarized in Table 2. The number of reproducible RFs varied largely from 91.4% (362 of 396, G.0, kiwis) to only 37.1% (147 of 396, G.1, volunteers) when the TR was modified by using CV index with 0.15 and 0.1 as the cutoff value, respectively. For each group of acquisition parameters (TR, TE, ST or AM), less than 50% RFs were reproducible in all groups of volunteers based on CV < 0.1. Moreover, we observed images with larger AM, thicker ST, shorter TE, or longer TR had more reproducible RFs, though there was no significant difference (p > 0.05) (Supplementary Materials Table S1).

**Table 2:** The number of reproducible features for intra-MR analysis with different acquisition parameters for volunteers and kiwis out of a total of 396.

Group		n	Number of reproducible features (%)		Mean ± standard deviation
			CV < 0.1	CV < 0.15	
**AM**					
Kiwis	G.0	3	290(73.2%)	324(81.8%)	0.12 ± 0.29
Volunteers	G.1	3	186(47.0%)	231(58.3%)	0.31 ± 1.41
	G.2	3	166(41.9%)	196(49.5%)	0.23 ± 0.65
	G.3	3	185(46.7%)	221(55.8%)	0.25 ± 0.47
**ST**					
Kiwis	G.0	3	324(81.85)	351(88.6%)	0.09 ± 0.19
Volunteers	G.1	3	193(48.7%)	236(59.6%)	0.24 ± 0.66
	G.2	3	190(48.0%)	239(60.4%)	0.18 ± 0.24
	G.3	3	197(49.7%)	238(60.1%)	0.23 ± 0.83
**TE**					
Kiwis	G.0	3	212(53.5%)	329(83.1%)	0.14 ± 0.22
Volunteers	G.1	3	172(43.4%)	227(57.3%)	0.32 ± 0.74
	G.2	3	154(38.9%)	204(51.5%)	0.24 ± 0.33
	G.3	3	183(46.2%)	208(52.5%)	0.23 ± 0.35
**TR**					
Kiwis	G.0	3	356(89.9%)	362(91.4%)	0.08 ± 0.23
Volunteers	G.1	3	147(37.1%)	219(55.3%)	0.27 ± 0.71
	G.2	3	194(49.0%)	214(54.0%)	0.22 ± 0.72
	G.3	3	157(39.6%)	201(50.7%)	0.35 ± 0.73

*TR* repetition time; *TE* echo time; *ST* slice thickness; *AM* acquisition matrix; G.0 represents the three kiwis, while G.1–G.3 represent the three group volunteers in 6th–10th, 11th–15th, and16th–20th day of physiological cycle respectively; The values displayed on the table were means within each group. “Mean ± Standard Deviation” was calculated from mean CV values within each group. MR scanner: Philips Medical Systems (Ingenia 3.0 T, Philips Healthcare, Best, The Netherlands).

### Feature selection and effects of intensity normalization

**Figure 1:**
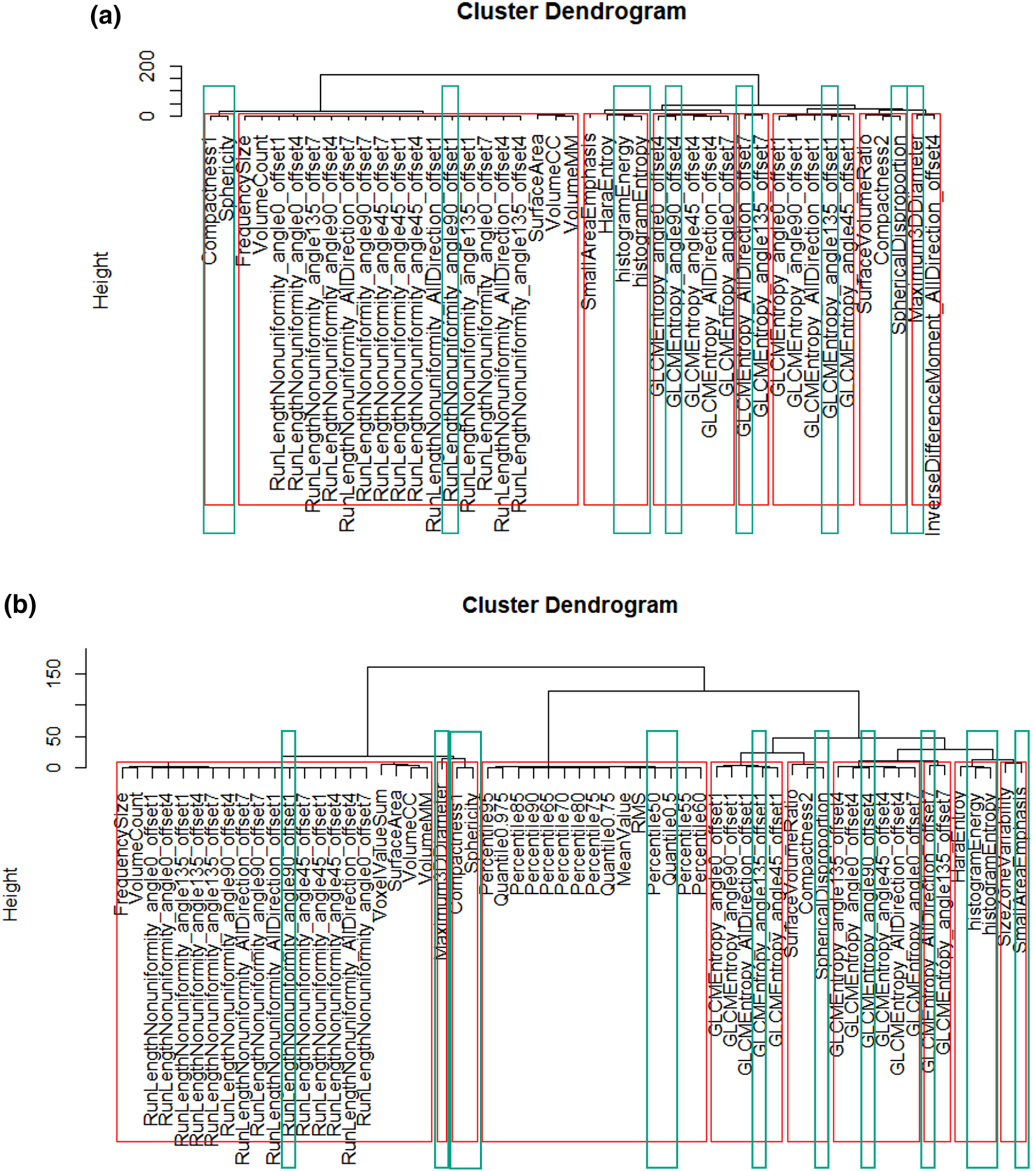
Hierarchical cluster analysis. *Note* Cluster dendrograms without intensity normalization (**a**) and with intensity normalization (**b**). The red frames represent different groups and within them the green frames highlight the representative features selected out.

**Figure 2:**
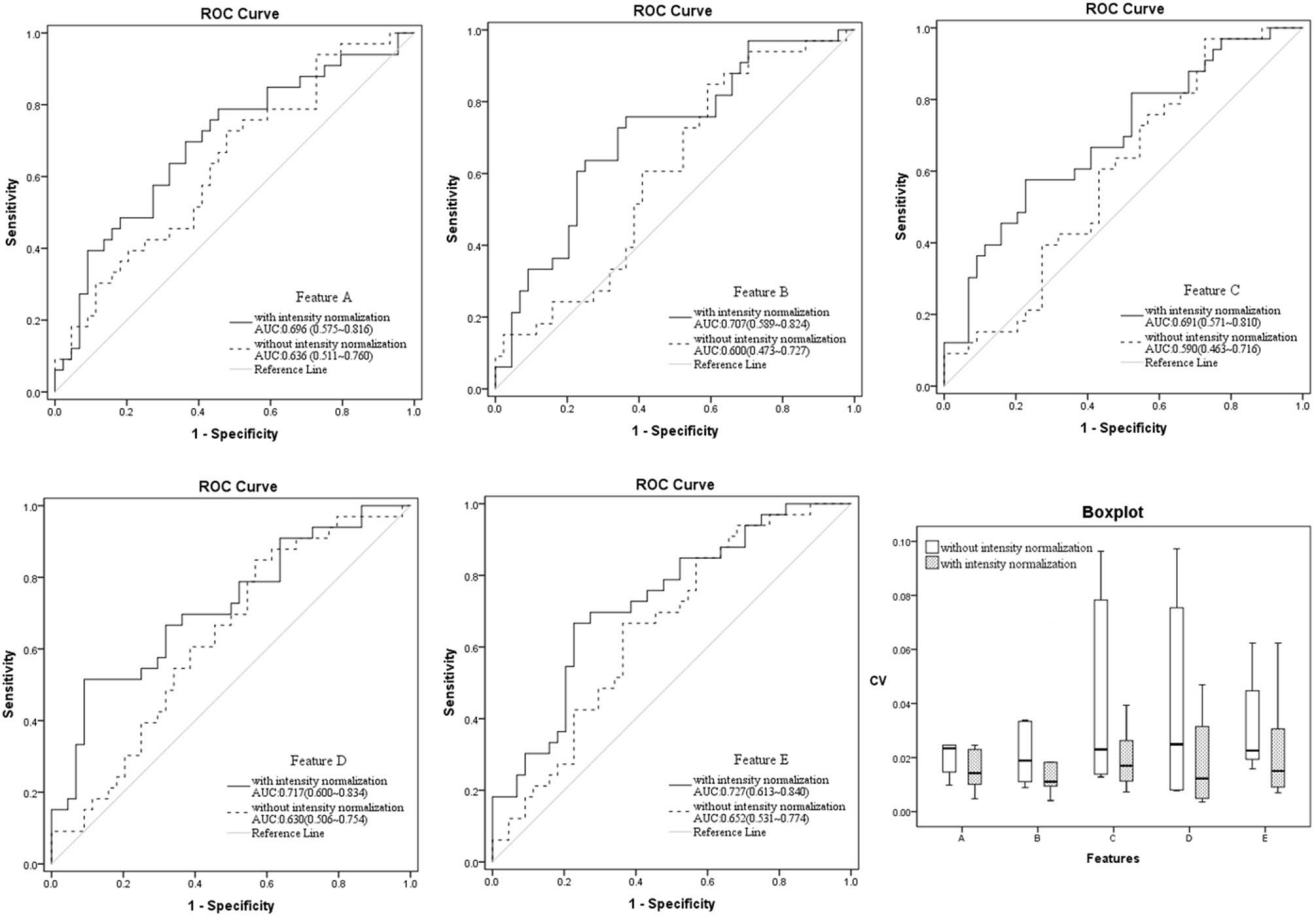
ROC curves and the boxplot. *Note* From ROC curves, all the area under the ROC curve values of these representative radiomics features in discriminating cervical junctional zone from outer muscular layer in leiomyoma patients were higher with intensity normalization than those without intensity normalization. And the boxplot showed that all the CV values were lower with intensity normalization than those without intensity normalization among these common representative radiomics features. Feature A: GLCMEntropy_angle90_offset4; Feature B: GLCMEntropy_AllDirection_offset7; Feature C: GLCMEntropy_angle135_offset1; Feature D: RunLengthNonuniformity_angle90_offset1; Feature E: histogram Energy/Entropy.

Based on CV < 0.1 and QCD < 10, we obtained 43 reproducible features in both inter-MR and intra-MR analyses for each kiwi and each volunteer including 4 histograms, 9 Form Factors, 14 GLCM, 15 RLM and 1 GLZSM features without intensity normalization (Supplementary Materials Fig. S1). After hierarchical cluster analysis, 8 representative features were acquired according to the CV value in the volunteers’ inter-MR analysis, including Compactness1/ Sphericity (Form Factor), Spherical Disproportion (Form Factor), GLCM Entropy_angle90_offset4, GLCM Entropy_AllDirection_offset7, GLCM Entropy_angle135_offset1, histogram Energy/ histogram Entropy, Run Length Nonuniformity_angle90_offset1, and Maximum 3D Diameter (Form Factor) (Fig. 1a). With image intensity normalization, 60 reproducible features were obtained, with histogram features showing the greatest increase (from 4 to 20) (Supplementary Materials Fig. S1). Next, we selected 10 representative features after hierarchical cluster analysis, including Small Area Emphasis (GLZSM) and Percentile50/Quantile0.5 (histogram), with the remaining eight representative features were the same as without intensity normalization (Fig. 1b). Among these common representative RFs with and without intensity normalization, lower CV values were obtained with intensity normalization, and the area under the ROC curve values of these representative features in discriminating cervical junctional zone from outer muscular layer in leiomyoma patients were higher with intensity normalization (with vs without, 0.691–0.727 [95% CI 0.571–0.840] vs 0.590–0.652 [95% CI 0.463–0.774], respectively) (Fig. 2). Geometric features were not taken into account in this part owing to their unchanging nature between with and without intensity normalization.

## Discussion

In this study, we evaluated the reproducibility of radiomics features across different MR scanners and scanning parameters. We found that a large portion of RFs were non-reproducible, in both inter-and intra-MR analyses. The reproducibility and the discriminative power of RFs were both improved with intensity normalization.

A previous study analyzed the influence of CT scanners and acquisition parameters on reproducibility of RFs based on non-biological phantoms^12^. However, results observed in the non-biological phantom might not be applicable on human images after similar experiments. Different from analysis solely based on non-biological phantoms, our results that were based on kiwi phantoms and real human tissue can indicate reality of clinical radiomics. In this observational study, a smaller number of reproducible features were acquired from volunteers than that from refresh kiwis. The stable kiwis can be used to overcome the intrinsic impairment due to the anatomy, positioning or physiological change of cervix. The natural degeneration of kiwis could be ignored since the whole scanning process across the three scanners maximally lasted for two hours. Beyond that, the kiwi is rich in water and has naturally structured textures, which can produce good T2W images and appears suitable to be used to compare different MR protocols and scanners^21,22^. Therefore, the kiwi was utilized as the reference of feature selection and intensity normalization.

This preliminary study included scanning acquisition settings similar to what might be seen in patient scans. If the variability was found to be small, then the scanning protocol could serve as a baseline for future patient studies. Nevertheless, our study showed the quite severe variability of the features even based on consistent scanning parameters across different scanners, which might be caused by the difference in fundamental design of the scanners. The percentage of reproducible RFs obtained from inter-scanner analysis was lower than that from intra-scanner analysis, accordant with the previous study^12^. We also found that signal intensity varied greatly across the three scanners in this study, which cannot be addressed by unifying MR scanning parameters.

In routine MR diagnostic studies, there is a large variability in thickness of slices, pixel size of the images, TR, TE, echo train length or bandwidth resulting from user preferences, protocol requirements, manufacturer’s settings, etc. These parameters determine the voxel size, grey level and signal to noise ratio. Therefore, evaluating their impacts on MR radiomics features is of paramount importance. In this study, we found that all four parameters, AM, ST, TE, and TR can impact reproducibility of radiomics features. We also found that bigger AM, thicker ST, shorter TE and longer TR produced more reproducible RFs, though there was no significant difference. However, texture features of all categories are increasingly sensitive to acquisition parameter variations with increasing spatial resolution (bigger AM) unless the spatial resolution is sufficiently high^13^. Besides, thinner slice images acquired better diagnostic performance than thicker slice(thicker ST) images, which might be caused by larger partial volume effect for thicker slice images^23^. Thus, a future study focusing on balancing the reproducibility and diagnostic performance might be necessary.

The large variation in signal intensity across different scanners calls for calibration attempts. Intensity normalization is a pre-processing step in the MR radiomics analysis and is vital for successful deep learning-based MR image synthesis^24^, especially for non-quantitative images in a multi-center scenario for shrinking intensity difference. Various intensity normalization methods have been proposed, including Z-score, piecewise linear histogram matching (the decile method), fuzzy C-means based, Gaussian mixture model based, kernel density estimate based, whitestripe and so on, which have met with varying degrees of success and also have their respective limitations^24,25^. Discussing all of them is beyond the scope of this research, that is, impact of different scanners and acquisition parameters on robustness of MR radiomics features. Although Z-score is used in many radiomics studies, but this method mainly emphasizes standardizing data and make them comparable, which does not change the gray distribution histogram of images. Instead, the decile method^26^ can adjust the distribution of the intensity, which is useful to not only produce consistent images but maintain the difference between different tissues across different scanners and scan parameters. In this study, we chose the decile method for evaluation also owing to its ease of computation, customizability and speed while maintaining high accuracy, which has been verified in brain across a multi-site multi-scanner MRI data^25^. We demonstrated the effectiveness of the decile approach in cervix for shrinking intra- and inter-scanner variations while at the same time improving the ability for stratifying tissues in this study too.

There are several limitations in this study. First, we just used those established and most common radiomics features, excluding wavelet and Laplacian of Gaussian transformations. To our best knowledge, before deriving these filtered features we have to engage super-parameters, such as convolution kernel size, but no standard kernel size has been provided so far. Besides, the most reproducible were among those calculated on the non-transformed images while filtered features showed the biggest discrepancy^27^. Thus, analysis based on non-transformed images could achieve the purpose of this study instead of exhaustively testing all the image features. Second, only three MR scanners and just 3 T field strength were used. However, our preliminary study quantitatively showed some objective factors affecting MR radiomics’ application in a real-world medical scenario. Lastly, just the T2WI sequence was evaluated in this study. Other commonly used modalities, such as the apparent diffusion coefficient map, could be further investigated in future.

In conclusion, only a few RFs derived from T2WI were robust across different MR scanners and acquisition parameters based on females’ cervix, which might be improved by decile intensity normalization method.

## Methods

### Phantoms (Kiwis)

**Figure 3:**
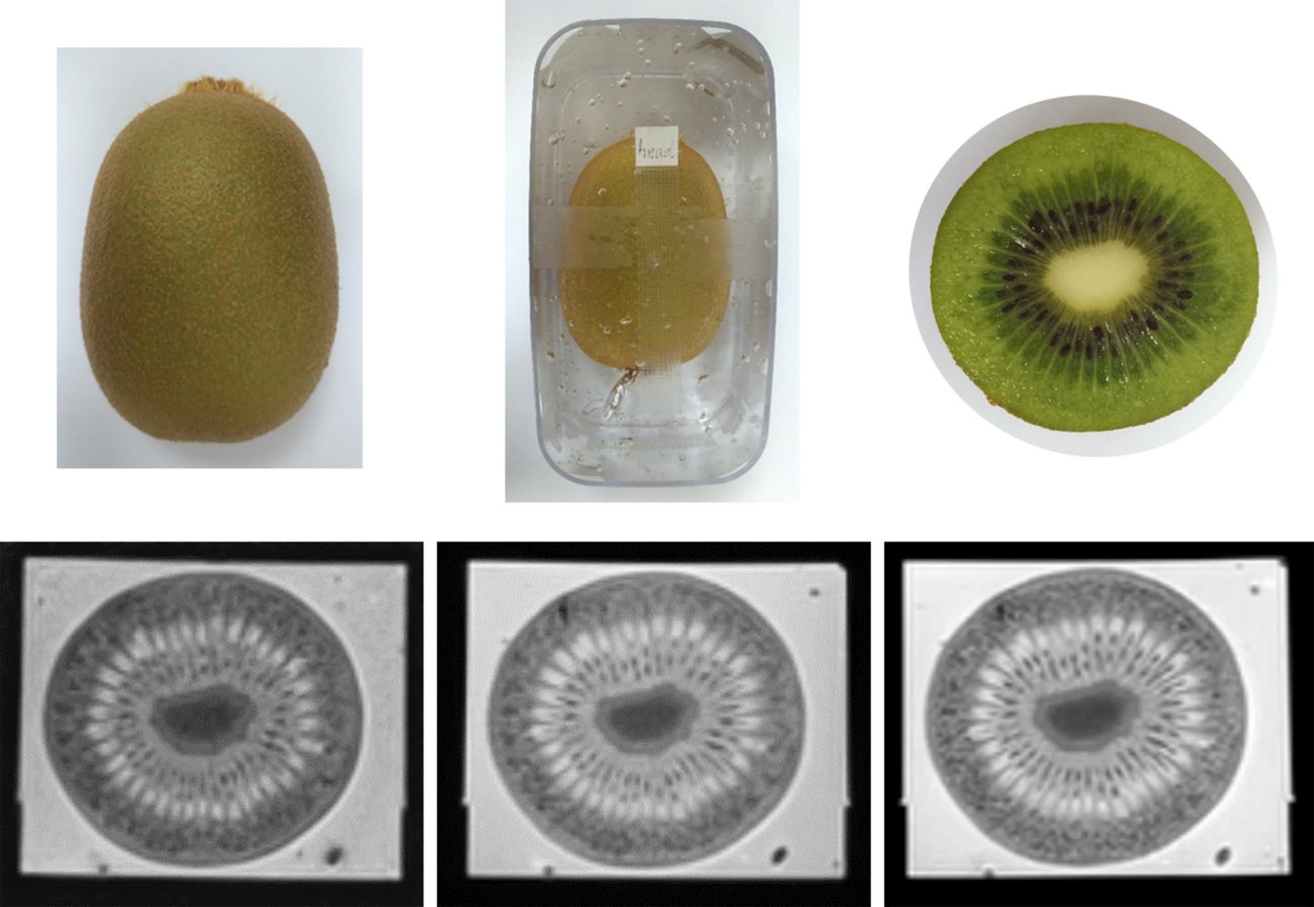
Kiwi phantom. *Note* Remove the hair of the green kiwi firstly, and then keep it in ultrasound gel within a tough plastic box of suitable size. T2 weighted images of a kiwi from three 3-T MR scanners are displayed below (Siemens, GE and Philips, respectively). Their scanning parameters were: 3000 ms (repetition time), 80 ms (echo time), 5 mm (slice thickness), 350 × 350 (field of view), 256 × 256 (acquisition matrix).

Prior to volunteers’ test, we performed a phantom examination as the reference of identifying reproducible RFs and image intensity normalization across multi-scanners and non-uniform scanning parameters. The phantom was selected based on the following criteria: biological, rich in water, suitable size (approximately 3 cm × 4 cm × 5 cm), certain degree of hardness, and stable textural characteristics. Kiwis were suitable for these criteria and three of them (green varietals, volume 70–75 cm^3^, NESPAR, Greece) were selected and characterized as group 0 (G.0) (Fig. 3). These kiwis were kept in thermostat at 7 °C before and between the experiments.

### Volunteers

This prospective observational study of healthy women was aimed to identify robust RFs across three different scanners and non-uniform scanning parameters within one scanner, which was approved by the Institutional Review Board of Renji Hospital, Shanghai Jiao Tong University School of Medicine, and the written informed consent was obtained from all volunteers before the MRI examinations. All procedures were performed in accordance with relevant guidelines and regulations. The inclusion criteria were healthy women with regular menstrual cycles (24–35 days)^28^ and negative gynecologic examination findings (gynecologic ultrasonography, serum tumor markers, cytology detection, and HPV DNA detection). A total of 9 women were included in our study (mean age, 25 years old; age range, 22–30 years). Considering that menstruation cycle could affect manifestation of cervix, thus, volunteers were divided into three groups according to their stage of menstrual cycle. Volunteers at 6th–10th, 11th–15th, and 16th–20th day (the date was calculated from the first day the participants had their regular bleeding) of physiological cycle were assigned as group 1, 2, 3, (G.1, G.2, G.3), respectively, with each group having three participants.

### Leiomyoma patients with healthy cervix

The Institutional Review Board of Renji Hospital, Shanghai Jiao Tong University School of Medicine, also approved the retrospective assessment on leiomyoma patients with healthy cervix with a waiver of informed consent. It aimed to estimate the robust RFs’ discriminative performance between the junctional zone and the outer muscular layer of healthy cervix in patients with leiomyoma in the body of the uterus. All procedures were performed in accordance with relevant guidelines and regulations. Sixty cases with negative results of cytology of cervical mucosa, gynecologic ultrasonography, serum tumor markers, and HPV DNA detection were enrolled consecutively during May, 2017 and April, 2019. Two cases were excluded in this study because of the image artifacts.

### MR data acquisition

The scanning parameters of leiomyoma patients were showed in Table 3. Their T2-weighted images were obtained with scanners from three different scanners, including GE Medical Systems (Signa HDxt 3.0 T, GE Healthcare, Wisconsin, USA) (n = 18), Philips Medical Systems (Ingenia 3.0 T, Philips Healthcare, Best, The Netherlands) (n = 21), and Siemens Medical Systems (Skyra 3.0 T, Siemens Healthcare, Erlangen, Germany) (n = 19). These systems were the most commonly used in radiomics studies on cervical lesions^5–7,18,19,29–33^. Thus, kiwis and volunteers were also scanned on these three scanners in the current study. To simulate the clinic reality, scanning protocols of kiwis and volunteers were referred to the clinical scanning parameters of leiomyoma patients.

**Table 3:** MR scan acquisition parameters.

Parameter (T2WI)	TR (ms)	TE (ms)	ST (mm)	Slice gap (mm)	Slice number	FOV (mmxmm)	ETL	AM	RM
**Clinical scanning (PH, GE, SI) (58 leiomyoma patients)**									
	3000–5000	80–110	3–6	0–1.5	13–25	320–383 × 224–323	15–23	256–380 × 256–360	240–392 × 240–392
**Inter-MR (PH, GE, SI) (3 kiwis and 9 volunteers)**									
	**3000**	**80**	**5**	0	15	350 × 350	15	**256 × 256**	512 × 512
**Intra-MRI (PH) (3 kiwis and 9 volunteers)**									
1: TR	**3000**	80	5	0	15	350 × 350	15	320 × 360	512 × 512
	**4000**	80	5	0	15	350 × 350	15	320 × 360	512 × 512
	**5000**	80	5	0	15	350 × 350	15	320 × 360	512 × 512
2: TE	3000	**80**	5	0	15	350 × 350	15	320 × 360	512 × 512
	3000	**90**	5	0	15	350 × 350	15	320 × 360	512 × 512
	3000	**100**	5	0	15	350 × 350	15	320 × 360	512 × 512
	3000	**110**	5	0	15	350 × 350	15	320 × 360	512 × 512
3: ST	3000	80	**3**	0	25	350 × 350	15	320 × 360	512 × 512
	3000	80	**4**	0	20	350 × 350	15	320 × 360	512 × 512
	3000	80	**5**	0	16	350 × 350	15	320 × 360	512 × 512
	3000	80	**6**	0	13	350 × 350	15	320 × 360	512 × 512
4: AM	3000	80	5	0	15	350 × 350	15	**256 × 256**	512 × 512
	3000	80	5	0	15	350 × 350	15	**320 × 256**	512 × 512
	3000	80	5	0	15	350 × 350	15	**320 × 360**	512 × 512
	3000	80	5	0	15	350 × 350	15	**380 × 280**	512 × 512

*TR* repetition time; *TE* echo time; *ST* slice thickness; *FOV* field of view; *ETL* echo train length; *AM* acquisition matrix; *RM* reconstruction matrix; *PH* Philips Medical Systems; *GE* GE Medical Systems; *SI* Siemens Medical Systems.

**Figure 4:**
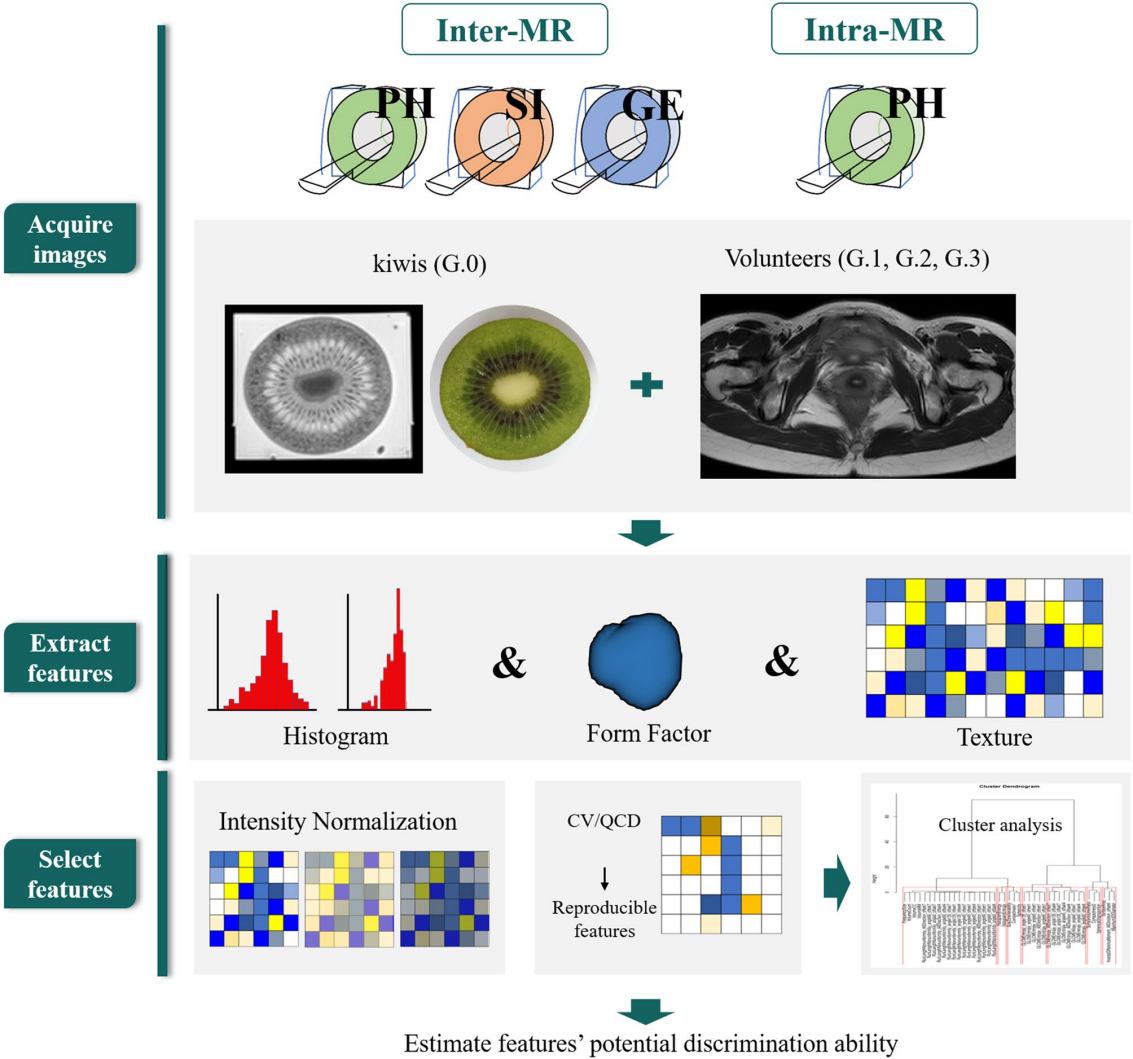
Study workflow. *Note* “Form Factor” means geometric feature; *PH* Philips Medical Systems; *GE* GE Medical Systems; *SI* Siemens Medical Systems; *G.0–G.3* group 0–group 3. *CV* the coefficient of variation, *QCD* the quartile coefficient of dispersion.

The whole study workflow of volunteers and kiwi-phantoms was showed in Fig. 4. For inter-MR process, we adjusted scanning parameters to be consistent across the three MR scanners. Each volunteer and kiwi-phantom was scanned sequentially with a short interval (less than 30 min) among scanners with a dedicated phased-array abdominal coil. For intra-MR process, four groups of parameters were modified only in Philips Medical System (Ingenia 3.0 T, Philips Healthcare, Best, The Netherlands): repetition time (TR: (1) 3000 ms, (2) 4000 ms, (3) 5000 ms), echo time (TE: (4) 80 ms, (5) 90 ms, (6) 100 ms, (7) 110 ms), slice thickness (ST: (8) 3 mm, (9) 4 mm, (10) 5 mm, (11) 6 mm), acquisition matrix (AM: (12) 256 × 256, (13) 320 × 256, (14) 320 × 360, (15) 380 × 280). During the experiment one acquisition parameter was changed at one scanning session while the rest of the parameters were kept constant. Then we changed another parameter after the former parameter scanning finished. Fifteen sequences ((1)–(15)) of images were acquired on each kiwi and volunteer. Details are showed in Table 3.

We performed T2 weighted imaging without fat suppression for both kiwis and volunteers and the whole scanning process was less than two hours for each of them. When acquiring kiwi’s axial images, we used a house-made adaptive holder to fix kiwi within ultrasound gel to immobilize the phantom during scanning (Fig. 3). Especially, volunteers were asked to fast for 4–6 h and receive butylscopolamine bromide intramuscularly (20 mg) before scanning in each scanner to avoid variation of cervix caused by intestinal movement. The scanning orientation of volunteers was based on cervix’s major axis, including parallel (sagittal) and vertical (axial) plane. The vertical plane crossing the margin of cervical opening was taken as baseline.

### Image preprocessing

A preprocessing pipeline was applied on all T2-weighted images, including the bias field correction, isotropic voxel resampling, registration, intensity normalization and gray-level discretization. To identify the effects of intensity normalization, data with and without intensity normalization was acquired separately.

First, the bias field correction was performed by using N4ITK for all images^34^. And then volumetric regions were isotopically resampled to the in-plane resolution (voxel size = 1mmx1mmx1mm) using cubic interpolation. Third, co-registration^35,36^ via SPM12 (https://www.fil.ion.ucl.ac.uk/spm/software/spm12/) was carried out in order to correct motion artifacts under different scanners or from a long scanning process in one scanner. Next, the decile based on piece-wise linear approach was used for intensity normalization^26,37^. To eliminate the high and unstable signal intensity of urine, the bladder tissue was excluded from images before normalization. Intensity normalization was performed by rescaling the intensity range of each input image (source) to match the referred image (reference) in Matlab software (https://www.mathworks.com). The grey value of the randomly selected reference was divided into 10 quantiles: 5%, 10%, 20%, 30%, 40%, 50%, 60%, 70%, 80%, 90%, and 95%. The minimum and maximum grey values were abandoned due to the noise effect. Regulated values were obtained using cubic interpolation. At last, the gray-level discretization inside the ROI was also applied to reduce the computational time and to improve the signal-to-noise ratio of the texture outcome^38^. This discretization step was built in the Artificial Intelligent Kit (A.K.) offered by GE Healthcare. The ROI data was initially decimated to 256 Gy levels via histogram equalization before extracting features.

### Regions of interest (ROIs)

**Figure 5:**
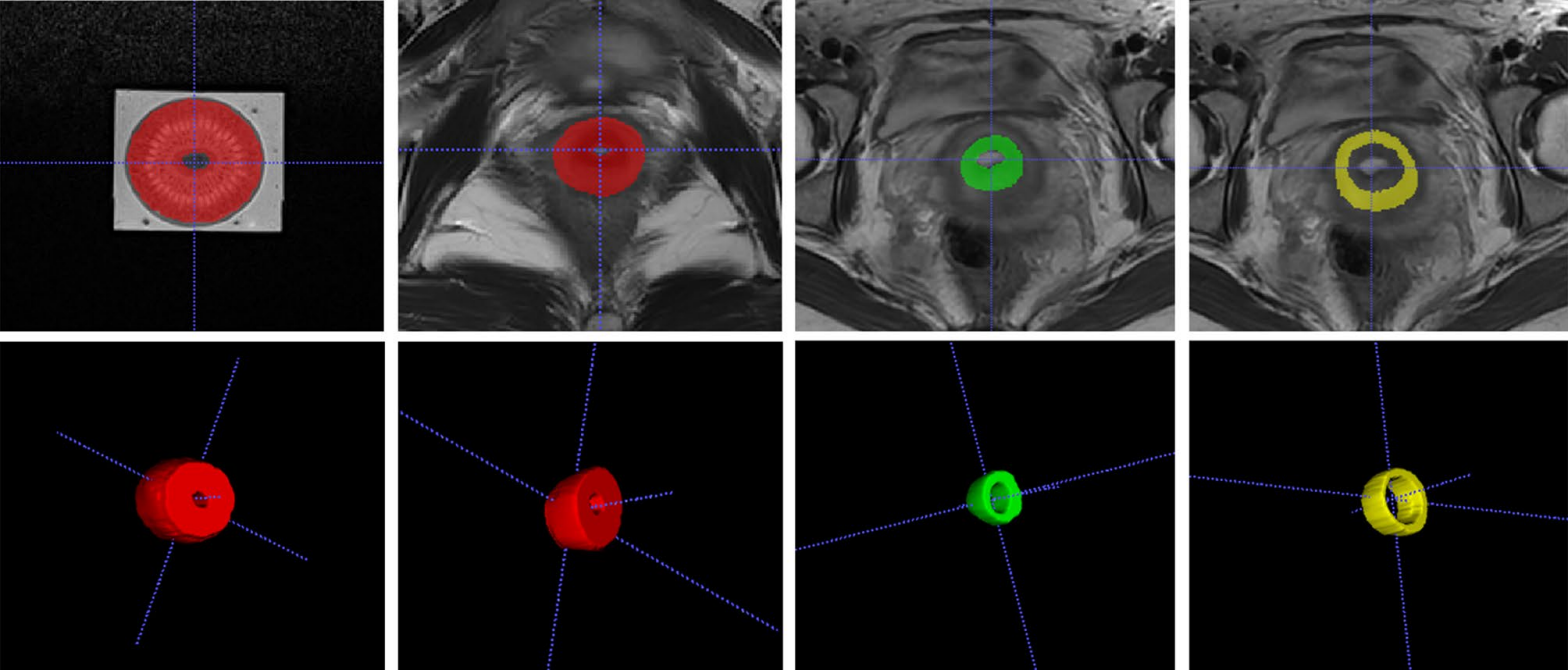
Delineation of three-dimensional regions of interest. *Note* Three-dimensional regions of interest of the kiwi covered almost the whole pulp of the central 5 slices of axial images except the skin and the central hypo-intensity region (**a**). To segment the normal cervix of volunteers, the whole cervix (**b**) including the endocervix, the junctional zone and the outer muscular layer was selected and liquid in cervical canal was excluded. For all leiomyoma patients enrolled in this study, the junctional zone (**c**) and the outer muscular layer (**d**) of their healthy cervix were delineated separately.

For each kiwi and each volunteer, the regions of interest (ROIs) of images from Philips and one sequence of parameters (3000 ms TR, 80 ms TE, 5 mm ST, 350 × 350 FOV, 256 × 256 AM) were firstly delineated manually on the ITK-SNAP software (https://www.itksnap.org) and then copied to images of the other scanners and other scanning parameters to avoid variations in segmentation. The ROIs of the kiwi covered almost the whole pulp of the central 5 slices of axial images except the skin and the central hypo-intensity region. To segment the normal cervix of volunteers, the whole body of cervix (including the endocervix, the junctional zone and the outer muscular layer of cervix) was selected and liquid in cervical canal was excluded. As for patients, the junctional zone and the outer muscular layer of their cervix were delineated separately (Fig. 5). All segmentations of ROIs were delineated by a junior radiologist with 4 years of experience in gynecological imaging firstly and then validated by a senior radiologist with 16 years of experience in gynecological imaging. Disagreement was resolved by consensus. The ROIs of each section were summated to derive a 3D volume of interest (VOI).

### Feature extraction

Images and corresponding VOIs were imported to the A.K. software. With the purpose of maximizing the comparability and common usability of features, we simplified the feature set from thousands to 396, including 42 histogram features, 9 geometric features (Form Factor) and 345 texture features. Histogram features represent the values of voxel intensity via first-order statistics^39^. For texture features, the rotation angles of an offset were 0°, 45°, 90°, and 135°. And the displacement vectors were the distance to the neighbor pixel: 1, 4, 7, different distributions from the same image of reference. Texture features mainly included 100 Gy-level co-occurrence matrix (GLCM), 180 Gy-level run-length matrix (RLM), 11 grey- level size zone matrix (GLSZM), 36 Cluster, and 18 Correlation features. Features were calculated within each VOI according to their definitions and formulas displayed in Supplementary Information.

### Variables and feature selection

The coefficient of variation (CV) was the main index used to evaluate the inter- and intra- MR reproducibility of RFs, and quartile coefficient of dispersion (QCD) was the supplementary index. We set cut-off values of 0.1 and 0.15 for CV, and 10 and 15 for QCD, to select reproducible RFs^12^. Their formulas are as follows:

1 $$CV = \frac{\sigma }{\mu },$$

defined as the ratio of the standard deviation to the mean^40^;

2 $$QCD = \frac{{Q_{3} - Q_{1} }}{{Q_{3} + Q}} \times 100,$$

where Q_1_ and Q_3_ are the first and third quartiles^41^, respectively. The selection workflow of representative robust RFs included five steps. First and second, inter- and intra- MR reproducible RFs were selected by CV < 0.1 and QCD < 10 for all the kiwis. Third and fourth, the inter-MR and intra-MR analysis of all the volunteers were performed to further select features from reproducible RFs after the second step by CV < 0.1 and QCD < 10. And finally, a hierarchical cluster analysis used for grouping similar features from these selected reproducible features in the fourth step was performed. In every cluster, the RF with the lowest CV value in the volunteers’ inter-MR analysis was taken as the representative robust RF. (Supplementary Materials Fig. S1).

### Statistical analysis

Statistical analysis was performed in R software v.3.5.0 (https://www.Rproject.org) and IBM SPSS software v.23. CV, QCD were calculated by DescTools. The hierarchical cluster analysis was done through the hclust and rect. hclust functions. Comparison of the higher value group and the lower value group in each sort of scanning parameters was using t-test over the mean CV values. p < 0.05 indicates statistical significance. Receiver operating characteristic curve (ROC) analysis was carried out to identify the capability of representative RFs in discriminating cervical junctional zone from outer muscular layer in leiomyoma patients with healthy cervix. Boxplot was used to show the difference of reproducibility of the representative features between with and without intensity normalization.

## Supplementary information

Supplementary Information.

## Data Availability

All data generated or analysed during this study are included in this published article (and its Supplementary Information files).

## References

[1] Lambin P (2012). Radiomics: extracting more information from medical images using advanced feature analysis. Eur. J. Cancer.

[2] Jiang X (2020). MRI based radiomics approach with deep learning for prediction of vessel invasion in early-stage cervical cancer. IEEE/ACM Trans. Comput. Biol. Bioinform..

[3] Hua W, Xiao T, Jiang X, Liu Z, Wang M, Zheng H, Wang S (2020). Lymph-vascular space invasion prediction in cervical cancer: Exploring radiomics and deep learning multilevel features of tumor and peritumor tissue on multiparametric MRI. Biomed. Signal Process. Control..

[4] Aerts HJ (2014). Decoding tumour phenotype by noninvasive imaging using a quantitative radiomics approach. Nat. Commun..

[5] Downey K (2013). Relationship between imaging biomarkers of stage I cervical cancer and poor-prognosis histologic features: quantitative histogram analysis of diffusion-weighted MR images. AJR Am. J. Roentgenol..

[6] Kan Y (2018). Radiomic signature as a predictive factor for lymph node metastasis in early-stage cervical cancer. J. Magn. Reson. Imaging.

[7] Liu Y (2018). Radiomics analysis of apparent diffusion coefficient in cervical cancer: A preliminary study on histological grade evaluation. J. Magn. Reson. Imaging.

[8] Zhang Z (2018). A predictive model for distinguishing radiation necrosis from tumour progression after gamma knife radiosurgery based on radiomic features from MR images. Eur. Radiol..

[9] Whitney HM (2019). Additive benefit of radiomics over size alone in the distinction between benign lesions and luminal a cancers on a large clinical breast MRI dataset. Acad. Radiol...

[10] Shiradkar R (2018). Radiomic features from pretreatment biparametric MRI predict prostate cancer biochemical recurrence: preliminary findings. J. Magn. Reson. Imaging.

[11] Kim H (2016). Impact of reconstruction algorithms on CT radiomic features of pulmonary tumors: analysis of intra- and inter-reader variability and inter-reconstruction algorithm variability. PLoS ONE.

[12] Berenguer R (2018). Radiomics of CT features may be nonreproducible and redundant: influence of CT acquisition parameters. Radiology.

[13] Mayerhoefer ME, Szomolanyi P, Jirak D, Materka A, Trattnig S (2009). Effects of MRI acquisition parameter variations and protocol heterogeneity on the results of texture analysis and pattern discrimination: an application-oriented study. Med. Phys..

[14] Li ZC (2018). Multiregional radiomics features from multiparametric MRI for prediction of MGMT methylation status in glioblastoma multiforme: a multicentre study. Eur. Radiol..

[15] Fetit AE (2018). Radiomics in paediatric neuro-oncology: a multicentre study on MRI texture analysis. NMR Biomed..

[16] Ginsburg SB (2017). Radiomic features for prostate cancer detection on MRI differ between the transition and peripheral zones: preliminary findings from a multi-institutional study. J. Magn. Reson. Imaging.

[17] Balleyguier C (2011). Staging of uterine cervical cancer with MRI: guidelines of the European Society of Urogenital Radiology. Eur. Radiol..

[18] Meng J (2018). Texture analysis as imaging biomarker for recurrence in advanced cervical cancer treated with CCRT. Sci. Rep..

[19] Wu Q (2018). Radiomics analysis of multiparametric MRI evaluates the pathological features of cervical squamous cell carcinoma. J. Magn. Reson. Imaging.

[20] Wu Q (2019). Radiomics analysis of magnetic resonance imaging improves diagnostic performance of lymph node metastasis in patients with cervical cancer. Radiother. Oncol..

[21] Mueller-Lisse UG (2017). Everyman's prostate phantom: kiwi-fruit substitute for human prostates at magnetic resonance imaging, diffusion-weighted imaging and magnetic resonance spectroscopy. Eur. Radiol..

[22] Guo XM, Xiao X, Wang GX, Gao RF (2013). Vascular anatomy of kiwi fruit and its implications for the origin of carpels. Front. Plant Sci..

[23] Zhao B, Tan Y, Tsai WY, Schwartz LH, Lu L (2014). Exploring variability in CT characterization of tumors: a preliminary phantom study. Transl. Oncol..

[24] Reinhold JC, Dewey BE, Carass A, Prince JL (2019). Evaluating the impact of intensity normalization on MR image synthesis. Proc. SPIE..

[25] Shah M (2011). Evaluating intensity normalization on MRIs of human brain with multiple sclerosis. Med. Image Anal..

[26] Nyul LG, Udupa JK, Zhang X (2000). New variants of a method of MRI scale standardization. IEEE Trans. Med. Imaging.

[27] Bogowicz M (2017). Post-radiochemotherapy PET radiomics in head and neck cancer: the influence of radiomics implementation on the reproducibility of local control tumor models. Radiother. Oncol..

[28] Speroff L, Mitchell C (1999). Part 1: Reproductive Physiology. Chapter 6: Regulation of the Menstrual Cycle. Clinical Gynecologic Endocrinology and Infertility.

[29] Becker AS (2017). MRI texture features may predict differentiation and nodal stage of cervical cancer: a pilot study. Acta Radiol Open.

[30] Guan Y (2016). Whole-lesion apparent diffusion coefficient-based entropy-related parameters for characterizing cervical cancers: initial findings. Acad. Radiol..

[31] Torheim T (2014). Classification of dynamic contrast enhanced MR images of cervical cancers using texture analysis and support vector machines. IEEE Trans. Med. Imaging.

[32] Lin Y (2015). Correlation of histogram analysis of apparent diffusion coefficient with uterine cervical pathologic finding. AJR Am. J. Roentgenol..

[33] Bowen SR (2018). Tumor radiomic heterogeneity: Multiparametric functional imaging to characterize variability and predict response following cervical cancer radiation therapy. J. Magn. Reson. Imaging.

[34] Tustison NJ (2010). N4ITK: improved N3 bias correction. IEEE Trans. Med. Imaging.

[35] Collignon A, Maes F, Delaere D, Vandermeulen D, Suetens P, Marchal G (1995). Automated multi-modality image registration based on information theory. Inf. Process. Med. Imaging.

[36] Ashburner J, Neelin P, Collins DL, Evans A, Friston K (1997). Incorporating prior knowledge into image registration. Neuroimage.

[37] Palumbo, D. *et al.* Interplay between bias field correction, intensity standardization, and noise filtering for T2-weighted MRI. *Conference proceedings: ... Annual International Conference of the IEEE Engineering in Medicine and Biology Society. IEEE Engineering in Medicine and Biology Society. Annual Conference* **2011**, 5080–5083, doi:10.1109/iembs.2011.6091258 (2011). 10.1109/IEMBS.2011.6091258 22255481

[38] Gibbs P, Turnbull LW (2003). Textural analysis of contrast-enhanced MR images of the breast. Magn. Reson. Med..

[39] Haralick RM, Shanmugam K, Dinstein I (1973). Textural features for image classification. IEEE Trans. Syst. Man Cybern..

[40] Reed GF, Lynn F, Meade BD (2002). Use of coefficient of variation in assessing variability of quantitative assays. Clin. Vaccine Immunol..

[41] Bonnet DG (2006). Confidence interval for a coefficient of quartile variation. Comput. Stat. Data. Anal..

