# Supplementary material for: Impact of different scanners and acquisition parameters on robustness of MR radiomics features based on women’s cervix
Source: Sci Rep. 2020 Nov 23;10:20407. doi: 10.1038/s41598-020-76989-0 (PMC7684312; doi:10.1038/s41598-020-76989-0)
Supplement: Supplementary file 1 — Supplementary Information. [file 41598_2020_76989_MOESM1_ESM.pdf]

Impact of Different Scanners and Acquisition Parameters on Robustness of MR Radiomics Features based on Women's Cervix

Honglan Mi[1], Mingyuan Yuan[2,+], Shiteng Suo[1], Jiejun Cheng[1], Suqin Li[1], Shaofeng Duan[3], Qing Lu[1,*]

Affiliations and addresses of the authors

[1] Department of Radiology, Renji Hospital, School of Medicine, Shanghai Jiao Tong University; 160 Pujian Rd, Shanghai, China. 200127.

[2] Department of Radiology, Affiliated Zhoupu hospital，Shanghai university of medicine & health Sciences College. 1500 Zhouyuan Road, PongDong New District, Shanghai, China. 201318

[3] GE Healthcare China, Pudong new town, No1, Huatuo road, Shanghai, 210000

[*] Corresponding authors: E-mail address: drluqingsjtu@163.com.

[+] This author contributed equally to this work.

Figure S1: Selected features shared across all the kiwis and all the volunteers in inter-MR and intra-MR analyses based on CV<0.1 and QCD<10. (with/without intensity normalization)

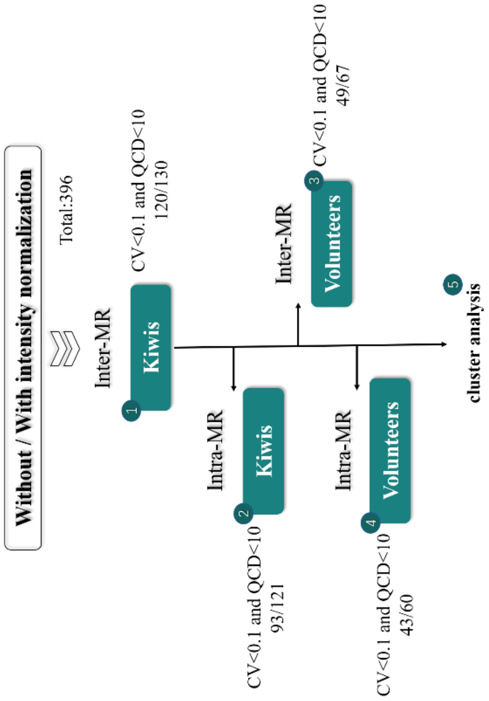

Total:	histogram :42		Form Factor:9		GLCM:100		RLM:180		GLZSM:11		Cluster:36		Correlation:18	
Reproducible features	without	with	without	with	without	with	without	with	without	with	without	with	without	with
	4	20	9	9	14	14	15	15	1	2	0	0	0	0
	FrequencySize	FrequencySize	Compactness1	Compactness1	GLCMEntropy_AllDirection_offset1	GLCMEntropy_AllDirection_offset1	RunLengthNonuniformity_AllDirection_offset1	RunLengthNonuniformity_AllDirection_offset1	SmallAreaEmphasis	SmallAreaEmphasis				
	VolumeCount	MeanValue	Compactness2	Compactness2	GLCMEntropy_AllDirection_offset4	GLCMEntropy_AllDirection_offset4	RunLengthNonuniformity_AllDirection_offset4	RunLengthNonuniformity_AllDirection_offset4		SizeZoneVariability				
	histogramEnergy	Percentile50	Maximum3DDiameter	Maximum3DDiameter	GLCMEntropy_AllDirection_offset7	GLCMEntropy_AllDirection_offset7	RunLengthNonuniformity_AllDirection_offset7	RunLengthNonuniformity_AllDirection_offset7						
	histogramEntropy	Percentile55	SphericalDisproportion	SphericalDisproportion	GLCMEntropy_angle0_offset1	GLCMEntropy_angle0_offset1	RunLengthNonuniformity_angle0_offset1	RunLengthNonuniformity_angle0_offset1						
		Percentile60	Sphericity	Sphericity	GLCMEntropy_angle0_offset4	GLCMEntropy_angle0_offset4	RunLengthNonuniformity_angle0_offset4	RunLengthNonuniformity_angle0_offset4						
		Percentile65	SurfaceArea	SurfaceArea	GLCMEntropy_angle0_offset7	GLCMEntropy_angle0_offset7	RunLengthNonuniformity_angle0_offset7	RunLengthNonuniformity_angle0_offset7						
		Percentile70	SurfaceVolumeRatio	SurfaceVolumeRatio	GLCMEntropy_angle135_offset1	GLCMEntropy_angle135_offset1	RunLengthNonuniformity_angle135_offset1	RunLengthNonuniformity_angle135_offset1						
		Percentile75	VolumeCC	VolumeCC	GLCMEntropy_angle135_offset7	GLCMEntropy_angle135_offset4	RunLengthNonuniformity_angle135_offset4	RunLengthNonuniformity_angle135_offset4						
		Percentile80	VolumeMM	VolumeMM	GLCMEntropy_angle45_offset1	GLCMEntropy_angle135_offset7	RunLengthNonuniformity_angle135_offset7	RunLengthNonuniformity_angle135_offset7						
		Percentile85			GLCMEntropy_angle45_offset4	GLCMEntropy_angle45_offset1	RunLengthNonuniformity_angle45_offset1	RunLengthNonuniformity_angle45_offset1						
		Percentile90			GLCMEntropy_angle90_offset1	GLCMEntropy_angle45_offset4	RunLengthNonuniformity_angle45_offset4	RunLengthNonuniformity_angle45_offset4						
		Percentile95			GLCMEntropy_angle90_offset4	GLCMEntropy_angle90_offset1	RunLengthNonuniformity_angle45_offset7	RunLengthNonuniformity_angle45_offset7						
		Quantile0.5			InverseDifferenceMoment_AllDirection_offset4	GLCMEntropy_angle90_offset4	RunLengthNonuniformity_angle90_offset1	RunLengthNonuniformity_angle90_offset1						
		Quantile0.75			HaraEntroy	HaraEntroy	RunLengthNonuniformity_angle90_offset4	RunLengthNonuniformity_angle90_offset4						
		Quantile0.975					RunLengthNonuniformity_angle90_offset7	RunLengthNonuniformity_angle90_offset7						
		RMS												
		VolumeCount												
		VoxelValueSum												
		histogramEnergy												
		histogramEntropy												

Table S1. The impact of scanner parameters on reproducibility of features based on volunteers' data. (totally 396 features)

Parameters	Range	Number of reproducible features (%)		p
		CV<0.1	CV<0.15	
AM	256x256~320x360	210 (53%)	239(60.4%)	0.468
	320x256~380x280	223(56.3%)	256(64.6%)	
ST (mm)	3~5	198(50.0%)	278(70.2%)	0.439
	4~6	210(53.0%)	299(75.5%)	
TE (ms)	80~100	192(48.5%)	254(64.1%)	0.302
	90~110	179(45.2%)	231(58.3%)	
TR (ms)	3000~4000	178(44.9%)	247(62.4%)	0.351
	4000~5000	200(50.5%)	266(67.2%)	

Note: TR: repetition time; TE: echo time; ST: slice thickness; AM: acquisition matrix. p values were calculated using t test based on 396 mean CV values of nine volunteers in each group. $p<0.05$ indicates different significantly. MR scanner: Philips Medical Systems (Ingenia 3.0T, Philips Healthcare, Best, The Netherlands).

Table S2. List of Radiomics Features.

Images and corresponding ROIs were imported to Artificial Intelligent Kit (A.K.), from which 42 histogram features, 9 geometric features (Form Factor) and 345 texture features were extracted out.

**categories**	**features**
histogram	FrequencySize
	MaxIntensity
	MeanDeviation
	MeanValue
	MedianIntensity
	MinIntensity
	Percentile5、10、15、20、25、30、35、40、45、50、55、60、65、70、75、80、85、90、95
	Quantile0.25、0.5、0.75、0.975
	RMS
	Range
	RelativeDeviation
	Variance
	VolumeCount
	VoxelValueSum

	histogramEnergy
	histogramEntropy
	Kurtosis
	Skewness
	stdDeviation
	Uniformity
Form Factor	Compactness1
	Compactness2
	Maximum3DDiameter
	SphericalDisproportion
	Sphericity
	SurfaceArea
	SurfaceVolumeRatio
	VolumeCC
	VolumeMM
GLCM	GLCMEnergy_AllDirection_offset1、4、7
	GLCMEnergy_AllDirection_offset1、4、7_SD
	GLCMEnergy_angle0_offset1、4、7
	GLCMEnergy_angle135_offset1、4、7
	GLCMEnergy_angle45_offset1、4、7
	GLCMEnergy_angle90_offset1、4、7

	GLCMEntropy_AllDirection_offset1、4、7
	GLCMEntropy_AllDirection_offset1、4、7_SD
	GLCMEntropy_angle0_offset1、4、7
	GLCMEntropy_angle135_offset1、4、7
	GLCMEntropy_angle45_offset1、4、7
	GLCMEntropy_angle90_offset1、4、7
	HaralickCorrelation_AllDirection_offset1、4、7
	HaralickCorrelation_AllDirection_offset1、4、7_SD
	HaralickCorrelation_angle0_offset1、4、7
	HaralickCorrelation_angle135_offset1、4、7
	HaralickCorrelation_angle45_offset1、4、7
	HaralickCorrelation_angle90_offset1、4、7
	Inertia_AllDirection_offset1、4、7
	Inertia_AllDirection_offset1、4、7_SD
	Inertia_angle0_offset1、4、7
	Inertia_angle135_offset1、4、7
	Inertia_angle45_offset1、4、7
	Inertia_angle90_offset1、4、7
	InverseDifferenceMoment_AllDirection_offset1、4、7
	InverseDifferenceMoment_AllDirection_offset1、4、7_SD
	InverseDifferenceMoment_angle0_offset1、4、7

	InverseDifferenceMoment_angle135_offset1、4、7
	InverseDifferenceMoment_angle45_offset1、4、7
	InverseDifferenceMoment_angle90_offset1、4、7
	AngularSecondMoment
	HaraEntroy
	HaraVariance
	Contrast
	differenceEntropy
	differenceVariance
	inverseDifferenceMoment
	sumAverage
	sumEntropy
	sumVariance
RLM	GreyLevelNonuniformity_AllDirection_offset1、4、7
	GreyLevelNonuniformity_AllDirection_offset1、4、7_SD
	GreyLevelNonuniformity_angle0_offset1、4、7
	GreyLevelNonuniformity_angle135_offset1、4、7
	GreyLevelNonuniformity_angle45_offset1、4、7
	GreyLevelNonuniformity_angle90_offset1、4、7
	HighGreyLevelRunEmphasis_AllDirection_offset1、4、7
	HighGreyLevelRunEmphasis_AllDirection_offset1、4、7_SD

	HighGreyLevelRunEmphasis_angle0_offset1、4、7
	HighGreyLevelRunEmphasis_angle135_offset1、4、7
	HighGreyLevelRunEmphasis_angle45_offset1、4、7
	HighGreyLevelRunEmphasis_angle90_offset1、4、7
	LongRunEmphasis_AllDirection_offset1、4、7
	LongRunEmphasis_AllDirection_offset1、4、7_SD
	LongRunEmphasis_angle0_offset1、4、7
	LongRunEmphasis_angle135_offset1、4、7
	LongRunEmphasis_angle45_offset1、4、7
	LongRunEmphasis_angle90_offset1、4、7
	LongRunHighGreyLevelEmphasis_AllDirection_offset1、4、7
	LongRunHighGreyLevelEmphasis_AllDirection_offset1、4、7_SD
	LongRunHighGreyLevelEmphasis_angle0_offset1、4、7
	LongRunHighGreyLevelEmphasis_angle135_offset1、4、7
	LongRunHighGreyLevelEmphasis_angle45_offset1、4、7
	LongRunHighGreyLevelEmphasis_angle90_offset1、4、7
	LongRunLowGreyLevelEmphasis_AllDirection_offset1、4、7
	LongRunLowGreyLevelEmphasis_AllDirection_offset1、4、7_SD
	LongRunLowGreyLevelEmphasis_angle0_offset1、4、7
	LongRunLowGreyLevelEmphasis_angle135_offset1、4、7
	LongRunLowGreyLevelEmphasis_angle45_offset1、4、7

	LongRunLowGreyLevelEmphasis_angle90_offset1、4、7
	LowGreyLevelRunEmphasis_AllDirection_offset1、4、7
	LowGreyLevelRunEmphasis_AllDirection_offset1、4、7_SD
	LowGreyLevelRunEmphasis_angle0_offset1、4、7
	LowGreyLevelRunEmphasis_angle135_offset1、4、7
	LowGreyLevelRunEmphasis_angle45_offset1、4、7
	LowGreyLevelRunEmphasis_angle90_offset1、4、7
	RunLengthNonuniformity_AllDirection_offset1、4、7
	RunLengthNonuniformity_AllDirection_offset1、4、7_SD
	RunLengthNonuniformity_angle0_offset1、4、7
	RunLengthNonuniformity_angle135_offset1、4、7
	RunLengthNonuniformity_angle45_offset1、4、7
	RunLengthNonuniformity_angle90_offset1、4、7
	ShortRunEmphasis_AllDirection_offset1、4、7
	ShortRunEmphasis_AllDirection_offset1、4、7_SD
	ShortRunEmphasis_angle0_offset1、4、7
	ShortRunEmphasis_angle135_offset1、4、7
	ShortRunEmphasis_angle45_offset1、4、7
	ShortRunEmphasis_angle90_offset1、4、7
	ShortRunHighGreyLevelEmphasis_AllDirection_offset1、4、7
	ShortRunHighGreyLevelEmphasis_AllDirection_offset1、4、7_SD

	ShortRunHighGreyLevelEmphasis_angle0_offset1、4、7
	ShortRunHighGreyLevelEmphasis_angle135_offset1、4、7
	ShortRunHighGreyLevelEmphasis_angle45_offset1、4、7
	ShortRunHighGreyLevelEmphasis_angle90_offset1、4、7
	ShortRunLowGreyLevelEmphasis_AllDirection_offset1、4、7
	ShortRunLowGreyLevelEmphasis_AllDirection_offset1、4、7_SD
	ShortRunLowGreyLevelEmphasis_angle0_offset1、4、7
	ShortRunLowGreyLevelEmphasis_angle135_offset1、4、7
	ShortRunLowGreyLevelEmphasis_angle45_offset1、4、7
	ShortRunLowGreyLevelEmphasis_angle90_offset1、4、7
GLZSM	SizeZoneVariability
	HighIntensityEmphasis
	HighIntensityLargeAreaEmphasis
	HighIntensitySmallAreaEmphasis
	IntensityVariability
	LargeAreaEmphasis
	LowIntensityEmphasis
	LowIntensityLargeAreaEmphasis
	LowIntensitySmallAreaEmphasis
	SmallAreaEmphasis
	ZonePercentage

Cluster	ClusterProminence_AllDirection_offset1、4、7
	ClusterProminence_AllDirection_offset1、4、7_SD
	ClusterProminence_angle0_offset1、4、7
	ClusterProminence_angle135_offset1、4、7
	ClusterProminence_angle45_offset1、4、7
	ClusterProminence_angle90_offset1、4、7
	ClusterShade_AllDirection_offset1、4、7
	ClusterShade_AllDirection_offset1、4、7_SD
	ClusterShade_angle0_offset1、4、7
	ClusterShade_angle135_offset1、4、7
	ClusterShade_angle45_offset1、4、7
	ClusterShade_angle90_offset1、4、7
Correlation	Correlation_AllDirection_offset1、4、7
	Correlation_AllDirection_offset1、4、7_SD
	Correlation_angle0_offset1、4、7
	Correlation_angle135_offset1、4、7
	Correlation_angle45_offset1、4、7
	Correlation_angle90_offset1、4、7

Note: Texture features ending with 1, 4, or 7 mean the sampling pixel distance and they represent different features.

Formulas and Definitions of radiomics features in this study via the A.K.

1. Histogram

Histogram parameters are concerned with properties of individual pixels. Let X denote the three dimensional image matrix with N voxels and P the first order histogram divided by N_l discrete intensity levels. The following first order statistics were extracted:

1.1 Energy:

The energy feature measures the uniformity of the intensity level distribution. If the value is high, then the distribution is to a small number of intensity levels. Energy can be defined as:

$$\text{energy} = \sum_{i}^{N} X(i)^2$$

1.2 Entropy:

The entropy measures the randomness of the distribution of the coefficients values over the intensity levels. If the value of entropy is high, then the distribution is among more intensity levels in the image. This measurement is the inverse of energy. A simple image has low entropy while a complex image has high entropy. Entropy can be defined as:

$$\text{entropy} = -\sum_{i=1}^{N_l} P(i) \log_2 P(i)$$

1.3 MaxIntensity:

The maximum intensity value of X.

1.4 MinIntensity:

The minimum intensity value of X.

1.5 MeanValue:

The mean measures the average value of the intensity values.

$$\text{mean} = \frac{1}{N}\sum_{i}^{N} X(i)$$

1.6 MeanDeviation:

The mean of the deviations of all voxel intensities around the mean intensity value.

1.7 MedianIntensity:

The median intensity value of X.

1.8 Range:

The range of intensity values of X.

1.9 Root mean square (RMS):

$$\text{RMS} = \sqrt{\frac{\sum_{i}^{N} X(i)^2}{N}}$$

1.10 Standard deviation: stdDeviation

Is a measure that is used to quantify the amount of variation or dispersion of a set of data values.

$$\text{standard deviation} = \left(\frac{1}{N-1}\sum_{i=1}^{N}(X(i)-\overline{X})^2\right)^{1/2}$$

where $\overline{X}$ is the mean of X.

1.11 Uniformity:

$$\text{uniformity} = \sum_{i=1}^{N_l} P(i)^2$$

1.12 Variance:

Is the average of the squared differences from the Mean.

$$\text{variance} = \frac{1}{N-1}\sum_{i=1}^{N}(X(i) - \overline{X})^2$$

where $\overline{X}$ is the mean of X.

1.13 Volume Count

Describe the size of the ROI.

1.14 Voxel Value Sum

Represents the Sum calculations for voxels in the ROI.

1.15 RelativeDeviation

Let $\overline{X}$ denote the mean of a set of quantities X_i , then the relative deviation is defined by:

$$\frac{\Delta X_i}{\overline{X}} = \frac{|X_i - \overline{X}|}{\overline{X}}$$

1.16 Frequency Size

1.17 Quantiles

For a finite population of N equally probable values indexed 1, …, N from lowest to highest, the k-th q-quantile of this population can equivalently be computed via the value of:

$$I_p = N\,k/q$$

1.18 Percentiles

A percentile (or a centile) is a measure used in statistics indicating the value below which a given percentage of observations in a group of observations fall.The percentile, p%, of a distribution is defined as that value of the brightness a such that:

P(a) = p%. or equivalently: $\int_{-\infty}^{a} P(\alpha) = p\%$

1.19 Skewness

Represents the degree of asymmetric distribution in the image histogram, this means that in some distribution of data, the right and the left of the distribution are perfect mirror images of one another, the mean, median and mode are all measures of the center of a set of data. The Skewness of the data can be determined by how these quantities are related to one another.

$$\text{skewness} = \frac{\frac{1}{N}\sum_{i=1}^{N}(X(i)-\overline{X})^3}{\left(\sqrt{\frac{1}{N}\sum_{i=1}^{N}(X(i)-\overline{X})^2}\right)^3}$$

where $\overline{X}$ is the mean of X.

1.20 Kurtosis

Kurtosis is a measure of whether the data are heavy-tailed or light-tailed

relative to a normal distribution. That is, data sets with high kurtosis tend to have heavy tails, or outliers. Data sets with low kurtosis tend to have light tails, or lack of outliers. A uniform distribution would be the extreme case.

$$\mathrm{kurtosis} = \frac{\frac{1}{N}\sum_{i=1}^{N}(X(i) - \bar{X})^4}{\left(\sqrt{\frac{1}{N}\sum_{i=1}^{N}(X(i) - \bar{X})^2}\right)^2}$$

where $\bar{X}$ is the mean of X.

2. Form Factor

These group of features includes descriptors of the three-dimensional size and shape of the tumor region. Let in the following definitions V denote the volume and A the surface area of the volume of interest. We determined the following shape and size based features:

2.1 Sphericity:

$$\mathrm{sphericity} = \frac{\pi^{\frac{1}{3}}(6V)^{\frac{2}{3}}}{A}$$

2.2 Surface area:

The surface area is calculated by triangulation (i.e. dividing the surface into connected triangles) and is defined as:

$$A = \sum_{i=1}^{N}\frac{1}{2}|a_i b_i \times a_i c_i|$$

2.3 Compactness 1:

$$\mathrm{compactness\ 1} = \frac{V}{\sqrt{\pi}A^{\frac{2}{3}}}$$

2.4 Compactness 2:

$$\text{compactness } 2 = 36\pi \frac{V^2}{A^3}$$

2.5 Maximum 3D diameter:

The maximum three-dimensional tumor diameter is measured as the largest pairwise Euclidean distance, between voxels on the surface of the tumor volume.

2.6 Spherical disproportion:

$$\text{spherical disproportion} = \frac{A}{4\pi R^2}$$

Where R is the radius of a sphere with the same volume as the tumor.

Where N is the total number of triangles covering the surface and a, b and c are edge vectors of the triangles.

2.7 Surfacetovolumeratio:

$$\text{surface to volume ratio} = \frac{A}{V}$$

2.8 VolumeCC and VolumeMM.

The maximum 3D diameter, surface area and volume provide information on the size of the lesion. Measures of compactness, spherical disproportion, sphericity and the surface to volume ratio describe how spherical, rounded, or elongated the shape of the tumor is.

3. GLCM

The Grey level co-occurrence matrix (GLCM) $P\ (i, j|\theta, d)$ represents the joint probability of certain sets of pixels having certain grey-level values. It calculates how many times a pixel with grey-level i occurs jointly with another pixel having a grey value j. By varying the displacement vector d between each pair of pixels. The rotation angle of an offset: $0°, 45°, 90°, 135°$ and displacement vectors (distance to the neighbor pixel: 1, 4, 7), different co-occurrence distributions from the same image of reference. GLCM of an image is computed using displacement vector d defined by its radius, (distance or count to the next adjacent neighbor preferably is equal to one) and rotational angles.

3.1 Energy of GLCM

This feature Returns the sum of squared elements in the GLCM. Range = [0 1]

Energy is 1 for a constant image. Is high when image has very good homogeneity

or when pixels are very similar The Property Energy is also known as uniformity, uniformity of energy, and angular second moment.

$$\sum_{i,j} g(i, j)^2$$

*g is a GLCM

Where i,j are the spatial coordinates of g (i,j).

3.2 Entropy of GLCM

Entropy is a measure of randomness of intensity image.

$$-\sum_{i,j} g(i, j) \log_2(i, j)$$

3.3 Inertia of GLCM

It reflects the clarity of the image and texture groove depth.

$$\sum_{\mathbf{i,j}}((\mathbf{i}-\mathbf{j})^2\mathbf{g}(\mathbf{i},\mathbf{j}))$$

3.4 Inverse Difference Moment

Inverse Difference Moment (IDM) is the local homogeneity. It is high when local gray level is uniform and inverse GLCM is high.

$$\sum\sum\frac{\mathbf{1}}{\mathbf{1}+(\mathbf{i}-\mathbf{j})^2}\mathbf{g}(\mathbf{i},\mathbf{j})$$

3.5 Haralick features

3.5.1 Haralick Correlation

Measures the degree of similarity of the gray level of the image in the row or column direction. Represents the local grey level correlation, the greater its value, the greater the correlation;

$$-\sum_{i,j}\frac{(i,j)g(i,j)-\mu_t^2}{\sigma_t^2}$$

* where μ_t and σ_t are the mean and standard deviation of the row (or column, due to symmetry) sums.

3.5.2 Angular Second Moment

$$f_1=\sum_{i=1}^{N_g}\sum_{j=1}^{N_g}\left(\frac{P(i,j)}{R}\right)^2=\sum_i\sum_j p(i,j)^2$$

3.5.3 Contrast

The contrast feature, is a difference moment of the P matrix and is a measure of the contrast or the amount of local variations present in the image.

$$f_2 = \sum_{k=0}^{N_g-1} k^2 \left\{ \sum_{i=1}^{N_g} \sum_{j=1}^{N_g} \delta_{|i-j|,k} p(i,j) \right\} = \sum_{k=0}^{N_g-1} k^2 p_{x-y}(k)$$

3.5.4 Haralick Entropy

$$f_9 = -\sum_{i=1}^{N_g} \sum_{j=1}^{N_g} p(i,j) \log(p(i,j))$$

3.5.5 HaraVariance

$$f_4 = \sum_{i=1}^{N_g} \sum_{j=1}^{N_g} (i-\mu)^2 p(i,j)$$

3.5.6 sumAverage

$$f_6 = \sum_{i=2}^{2N_g} i p_{x+y}(i)$$

3.5.7 sumVariance

$$f_7 = \sum_{i=2}^{2N_g} (i - f_8)^2 p_{x+y}(i)$$

3.5.8 sumEntropy

$$f_8 = -\sum_{i=2}^{2N_g} p_{x+y}(i) \log(p_{x+y}(i))$$

3.5.9 differenceVariance

$$f_{10} = \text{variance of } p_{x-y}$$

3.5.10 differenceEntropy

$$f_{11} = -\sum_{i=0}^{N_g-1} p_{x-y}(i) \log(p_{x-y}(i))$$

3.5.11 inverseDifferenceMoment

$$f_5 = \sum_{i=1}^{N_g} \sum_{j=1}^{N_g} \frac{1}{1+(i-j)^2} p(i,j)$$

4. RLM

The grey level run-length matrix (RLM) $\mathbf{P_r(i,j \mid \theta)}$ is defined as the numbers of runs with pixels of gray level i and run length j for a given direction θ. RLMs is generated for each sample image segment having directions (0°,45°,90° &135°), then the following ten statistical features were derived:

4.1 Short Run Emphasis (18 Parameters)

$$SRE(\theta) = \frac{1}{n_r} \sum_{i=1}^{M} \sum_{j=1}^{N} \frac{p(i,j,\theta)}{j^2}$$

4.2 Long Run Emphasis (18Parameters)

$$LRE(\theta) = \frac{1}{n_r} \sum_{i=1}^{M} \sum_{j=1}^{N} p(i,j,\theta)\, j^2$$

4.3 Grey Level Non-uniformity(18Parameters)

$$GLN(\theta) = \frac{1}{n_r} \sum_{i=1}^{M} \left(\sum_{j=1}^{N} p(i,j,\theta)\right)^2$$

4.4 Run Length Non-uniformity(18Parameters)

$$RLN(\theta) = \frac{1}{n_r}\sum_{j=i}^{N}(\sum_{i=1}^{M} p(i,j,\theta))^2$$

4.5 Low Grey Level Run Emphasis(18Parameters)

$$LGRE(\theta) = \frac{1}{n_r}\sum_{j=i}^{N}\sum_{i=1}^{M}\frac{p(i,j,\theta)}{i^2}$$

4.6 High Grey Level Run Emphasis(18Parameters)

$$HGRE(\theta) = \frac{1}{n_r}\sum_{j=i}^{N}\sum_{i=1}^{M} p(i,j,\theta)i^2$$

4.7 Short Run Low Grey Level Emphasis(18Parameters)

$$SRLGE(\theta) = \frac{1}{n_r}\sum_{j=i}^{N}\sum_{i=1}^{M}\frac{p(i,j,\theta)}{i^2 j^2}$$

4.8 Short Run High Grey Level Emphasis(18Parameters)

$$SRHGE(\theta) = \frac{1}{n_r}\sum_{j=i}^{N}\sum_{i=1}^{M}\frac{p(i,j,\theta)i^2}{j^2}$$

4.9 Long Run Low Grey Level Emphasis(18Parameters)

$$LRLGE(\theta) = \frac{1}{n_r}\sum_{j=i}^{N}\sum_{i=1}^{M}\frac{p(i,j,\theta)j^2}{i^2}$$

4.10 Long Run High Grey Level Emphasis(18Parameters)

$$LRHGE(\theta) = \frac{1}{n_r}\sum_{j=i}^{N}\sum_{i=1}^{M} p(i,j,\theta)i^2 j^2$$

where n_r is the total number of runs and n_p is the number of pixels in the image.

5. GLZSM

The gray level Size Zone Matrix (SZM) is the starting point of Thibault

matrices. For a texture image f with N gray levels, it is denoted GSf(s, g) and provides a statistical representation by the estimation of a bivariate conditional probability density function of the image distribution values. It is calculated according to the pioneering Run Length Matrix principle: the value of the matrix GSf(s, g) is equal to the number of zones of size s and of gray level g. The resulting matrix has a fixed number of lines equal to N, the number of gray

levels, and a dynamic number of columns, determined by the size of the largest zone as well as the size quantization.

5.1 SizeZoneVariability

$$\frac{1}{Ng\times Lz}\sum_i\sum_j(jp(i,j)-\mu_j)^2$$

5.2 HighIntensityEmphasis

$$\sum_i\sum_j i^2p(i,j)$$

5.3 HighIntensityLargeAreaEmphasis

$$\sum_i\sum_j i^2j^2p(i,j)$$

5.4 HighIntensitySmallAreaEmphasis

$$\sum_i\sum_j\frac{j^2p(i,j)}{i^2}$$

5.5 IntensityVariability

$$\frac{1}{Ng\times Lz}\sum_i\sum_j(ip(i,j)-\mu_i)^2$$

5.6 LargeAreaEmphasis

$$\sum_i \sum_j j^2 p(i,j)$$

5.7 LowIntensityEmphasis

$$\sum_i \sum_j \frac{p(i,j)}{j^2}$$

5.8 LowIntensityLargeAreaEmphasis

$$\sum_i \sum_j \frac{j^2 p(i,j)}{i^2}$$

5.9 LowIntensitySmallAreaEmphasis

$$\sum_i \sum_j \frac{p(i,j)}{i^2 j^2}$$

5.10 SmallAreaEmphasis

$$\sum_i \sum_j \frac{p(i,j)}{j^2}$$

5.11 ZonePercentage

$$\sum_i \sum_j \frac{\sum_i \sum_j p(i,j)}{\sum_j j \sum_i p(i,j)}$$

6. Cluster

Cluster analysis or clustering is the task of grouping a set of objects in such a way that objects in the same group (cluster) are more similar (in some sense or another) to each other than to those in other groups (clusters). It is a common technique for statistical data analysis.

6.1 Cluster Shade

Cluster Shade in clustered shading, we group similar view samples according to

their position and, optionally, normal into clusters.

$$\sum_{i,j}((i-\mu)+(j-\mu))^3 g(i,j)$$

6.2 Cluster Prominence

Cluster Prominence is a measure of asymmetry of a given distribution, high values of this feature indicate that the symmetry of the image is low, in medical imaging low values of cluster prominence represent a smaller peak for the image grey level value and usually the grey level difference between the forms is small.

$$\sum_{i,j}((i-\mu)+(j-\mu))^4 g(i,j)$$

7. Correlation

Correlation measures the similarity of the grey levels in neighboring pixels, tells how correlated a pixel is to its neighbor over the whole image. Range = [-1 1]. Correlation is 1 or -1 for a perfectly positively or negatively correlated image.

$$-\sum_{i,j}\frac{(i-\mu)(j-\mu)g(i,j)}{\sigma^2}$$

Reference:

1. P. Mohanaiah, P. Sathyanarayana and L. GuruKumar. 2013. Image Texture Feature Extraction Using GLCM Approach, International Journal of Scientific and Research Publications, Volume 3, Issue 5.

2. NIST/SEMATECH e-Handbook of Statistical Methods,

http://www.itl.nist.gov/div898/handbook/.

3. Alnihoud J. 2012. Content-Based Image Retrieval System Based on Self Organizing Map, Fuzzy Color Histogram and Subtractive Fuzzy Clustering, International Arab Journal of Information Technology, vol. 9, no. 5, pp. 452- 458.

4. Balanda, Kevin P., and H. L. MacGillivray. 1988. Kurtosis: A Critical Review. The American Statistician 42(2), 111–119.

5. https://en.wikipedia.org/wiki/Kurtosis

6. Bland, J.M.; Altman, D.G. 1996. Statistics notes: measurement error. BMJ. 312 (7047): 1654.

7. http://thibault.biz/Research/ThibaultMatrices/GLSZM/GLSZM.html

8. Galloway, M.M. 1975. Texture analysis using gray level run lengths. Computer Graphics and Image Processing, 4(2), 172-179.

9. Chu, A. Sehgal, C.M. and Greenleaf, J.F. 1990. Use of gray value distribution of run lengths for texture analysis. Pattern Recognition Letters, 11(6), 415-419.

10. Dasarathy, B.V. and Holder, E.B. 1991. Image characterization based on joint gray level-run length distributions. Pattern Recognition Letters, 12(8), 497-502.

11. Thibault, G. et al. 2009. Texture indexes and gray level size zone matrix. Application to cell nuclei classification. In Pattern Recognition and Information Processing. Minsk, Belarus, 140-145.
